# Microbial community and metabolomic comparison of irritable bowel syndrome faeces

**DOI:** 10.1099/jmm.0.028126-0

**Published:** 2011-06

**Authors:** Kannan Ponnusamy, Jung Nam Choi, Jiyoung Kim, Sun-Young Lee, Choong Hwan Lee

**Affiliations:** 1 Department of Bioscience and Biotechnology and Bio/Molecular Informatics Center, Konkuk University, Seoul 143-701, Republic of Korea; 2 Department of Internal Medicine, Konkuk University Medical Center, Seoul 143-701, Republic of Korea

## Abstract

Human health relies on the composition of microbiota in an individual’s gut and the synthesized metabolites that may alter the gut environment. Gut microbiota and faecal metabolites are involved in several gastrointestinal diseases. In this study, 16S rRNA-specific denaturing gradient gel electrophoresis and quantitative PCR analysis showed that the mean similarity of total bacteria was significantly different (*P*<0.001) in faecal samples from patients with irritable bowel syndrome (IBS; *n* = 11) and from non-IBS (nIBS) patients (*n* = 8). IBS subjects had a significantly higher diversity of total bacteria, as measured by the Shannon index (*H′*) (3.36<*H′*<4.37, *P* = 0.004), *Bacteroidetes* and lactobacilli; however, less diversity was observed for *Bifidobacter* (1.7<* H′*<3.08, *P*<0.05) and *Clostridium coccoides* (0.9<* H′*<2.98, *P* = 0.007). In this study, no significant difference was found in total bacterial quantity (*P*>0.05). GC/MS-based multivariate analysis delineated the faecal metabolites of IBS from nIBS samples. Elevated levels of amino acids (alanine and pyroglutamic acid) and phenolic compounds (hydroxyphenyl acetate and hydroxyphenyl propionate) were found in IBS. These results were highly correlated with the abundance of lactobacilli and *Clostridium*, which indicates an altered metabolism rate associated with these gut micro-organisms. A higher diversity of *Bacteroidetes* and *Lactobacillus* groups in IBS faecal samples also correlated with the respective total quantity. In addition, these changes altered protein and carbohydrate energy metabolism in the gut.

## Introduction

Irritable bowel syndrome (IBS) is a functional disorder of the gastrointestinal (GI) tract that results in abdominal pain, altered bowel habits and irregular stool characteristics. IBS cases have been reported worldwide, and it has been shown that adults and adolescents are more prone to developing IBS. In western European and North America, 10–15 % of the population have IBS, and 6.6 % of the Korean population are affected (Han *et al.*, 2006). Although the aetiology of IBS has not yet been identified, it is believed that genetic, environmental, psycho-social and physiological factors, as well as an individual’s diet, may be responsible for IBS (Ohman & Simrén, 2007). In addition, recent studies have shown differences in the microbiota between IBS diarrhoea patients and healthy controls (Krogius-Kurikka *et al.*, 2009). Short-chain fatty acids were found to be at higher levels in diarrhoea-predominant IBS patients than in other IBS patients (Mortensen *et al.*, 1987).

Intestinal micro-organisms play various roles in human health such as complex food digestion, metabolizing drugs, detoxifying toxic compounds, producing essential vitamins and preventing colonization of pathogens. Most of the micro-organisms found in the GI tract are anaerobic bacteria, which are uncultivable under standard laboratory conditions. The type and number of bacteria in the GI tract varies depending on age, gender, geographical origin (Mueller *et al.*, 2006) and environmental factors, such as diet and dietary supplements (Kajander *et al.*, 2009). *Firmicutes *and *Bacteroidetes* are the dominant beneficial bacteria present in the normal human GI tract, and the latter was reported in lower numbers in constipation-predominant IBS patients (Rajilić-Stojanović *et al.*, 2007). A variety of factors determine the intestinal residential and transient species populations (Heavey & Rowland, 1999). Therefore, the composition and proportion of individual bacterial populations, as well as metabolites, are continuously changing; in addition, the bacterial community depends on diet. As the GI tract harbours complex microbial, digested and non-digested food materials, micro-organisms cannot respond independently. Rather, group-specific bacteria act as a consortium and coordinate other competing microbials as well as the host metabolism, either positively or negatively. The general estimation of diversity and the selected beneficial and predominant community size may be correlated with healthy and diseased GI tracts. Metagenomics-based studies on microbial genomes in the GI tract have provided a more thorough understanding of the indigenous micro-organisms and their contribution to IBS. PCR was previously combined with denaturing gradient gel electrophoresis (DGGE) to provide more detailed information on microbial composition and diversity, and their specific roles in IBS (Rajilić-Stojanović *et al.*, 2007).

Recent studies have shown that human symbiotic gut microbiota modulate the microbial phenotype and metabolites in faecal and urinary samples (Li *et al.*, 2008). Thus, altered faecal metabolites not only reflect the status of the gut microbiome community but also bridge the relationships between symbiotic microbes and health. GC-MS has been applied successfully to analyse and interpret multiparametric metabolic responses in living systems, and pathophysiological and environmental perturbations (Gao *et al.*, 2009; Scherling *et al.*, 2010). Multivariate statistical analysis by partial least-squares discriminant analysis (PLS-DA) has been employed to elucidate significant differences and to screen for potential biomarkers that could account for the variance of biological samples (Gao *et al.*, 2009).

Very little work has been conducted to compare the faecal microbial community and metabolites among IBS patients in the Korean population. A better understanding of the prevalent bacterial community and metabolites in faecal samples might be useful for either eliminating or restoring certain species to improve one’s health. Therefore, in the present study, we analysed and compared the indigenous faecal microbial community of IBS and non-IBS (nIBS) Korean subjects with faecal metabolites. The data presented here provide details about the complex GI bacterial shift and faecal metabolites, which could ultimately help delineate IBS from nIBS.

## Methods

### 

#### Study subjects.

Faecal samples were collected from 11 IBS (6 male, 5 female) and 8 nIBS (6 male, 2 female) patients, aged 18–74 years (mean 47.5 years) who were advised to follow a normal diet without any medications (see Supplementary Table S1 available with the online journal). The Rome II standard was followed to select the study subjects, and the exclusion criteria included inflammatory bowel disease (ulcerative colitis, Crohn’s disease and Behcet’s disease), GI malignancy and acute GI bleeding.

#### Bacterial community DNA extraction.

The collected faecal samples were frozen immediately (<5 min) at −20 °C and stored at −80 °C until used. Faecal community DNA was extracted as described in published studies (Apajalahti *et al.*, 1998) and purified using a PowerClean DNA clean-up kit (Mo Bio Laboratories). The DNA concentration and purity were determined using a Nanodrop spectrophotometer (Nanodrop Technologies).

#### PCR amplification of total bacteria.

Total bacterial DNA was used for the amplification of the V3–V5 region of the 16S rRNA gene using universal primers GC341F (341F with a GC clamp) and 907R as shown in Table 1. The PCR mixture (25 µl) contained 5 ng template DNA, 0.18 µM each primer, 0.25 mM dNTPs, 0.75 U *Ex Taq* polymerase (Takara) and reaction buffer with 2 mM MgCl_2_. The V3–V5 regions were amplified using GC clamp primers with an initial denaturation step of 95 °C for 5 min, followed by touchdown PCR: 9 cycles of 94 °C for 30 s, 64 to 56 °C for 28 s at a ramp of −1 °C per cycle and 72 °C for 50 s; and then 15 cycles of 94 °C for 30 s, 56 °C for 28 s and 72 °C for 50 s, with a final elongation at 72 °C for 15 min. Two microlitres of the amplicon was analysed by (1.2 %, w/v) agarose gel electrophoresis prior to DGGE analysis.

**Table 1.** Universal and group-specific 16S rRNA gene-targeted primers and PCR/DGGE conditions

Primer	Sequence (5′→3′)	Amplicon size (bp)	PCR	DGGE			Target group	Reference
			Annealing temp (°C)	Acrylamide (%)	Gradient (%)	Run		
341F	CCTACGGGAGGCAGCAGCCG*	585	56	6	36–54	90 V/16 h	Universal bacteria (16S rRNA gene variable regions V3–V5)	Muyzer & Smalla (1998)
907R	TCAATTCMTTTGAGTTT							
BfraF	GGTGTCGGCTTAAGTGCCAT	140	62	8	40–65	135 V/5 h	*Bacteroides–Prevotella–Porphyromonas*	Rinttilä *et al.* (2004)
BfraR	CGGA(C/T)GTAAGGGCCGTGC*							
BifF	GCGTGCTTAACACATGCAAGTC	125	60	8	40–70	135 V/5 h	Bifidobacteria	Zwielehner *et al.* (2009)
BifR	CACCCGTTTCCAGGAGCTATT*							
CoccoF	AAATGACGGTACCTGACTAA	440	53	6	38–58	70 V/16 h	*Clostridium coccoides*	Matsuki *et al.* (2002)
CoccoR	CTTTGAGTTTCATTCTTGCGAA*							
LacF	AGCAGTAGGGAATCTTCCA	380	56	8	38–53	70 V/16 h	Lactobacilli	Vanhoutte *et al.* (2004)
LacR	ATTYCACCGCTACACATG*							

F, Forward; R, reverse.

* GC clamp, CGCCCGGGGCGCGCCCCGGGCGGGGCGGGGGCACGGGGGG.

#### Group-specific bacteria amplification.

The composition of selected probiotic and predominant bacterial groups such as *Bacteroidetes*, *Bifidobacter*, *Clostridium coccoides* and *Lactobacillus* groups was studied using group-specific amplifications to compare the individual communities with the total bacteria. The primers and PCR conditions for group-specific amplification are described in Table 1.

#### DGGE.

DGGE was performed using a DCode universal mutation detection system (Bio-Rad). Briefly, PCR samples were applied to polyacrylamide gels in 1× TAE with a urea and formamide denaturing gradient [7 M urea and 40 % (v/v) formamide corresponded to the 100 % gradient] as described by Muyzer & Smalla (1998). Electrophoresis was carried out at 60 °C with a constant voltage, as described in Table 1. The gels were stained with SYBR Green I (Invitrogen), observed under UV transillumination and documented with a Molecular Imager GelDoc system (Bio-Rad). The PCR amplicons of type strains and a representative study sample were included as markers to provide a possible indication of the identities and to compare bands between the gels. The following type strains were obtained from the Korean Agricultural Culture Collection (KACC) or Korean Collection for Type Cultures (KCTC): *Bacteroides fragilis* (KCTC 3688), *Bacteroides vulgatus* (KCTC 2639), *Bifidobacterium breve* (KCTC 3220), *Bifidobacterium longum* (KACC 20597), *Campylobacter jejuni* (KCTC 5327), *Clostridium clostridioforme* (KCTC 5572), *Enterococcus faecalis* (KACC 13807), *Escherichia coli* (KCTC 1682), *Lactobacillus acidophilus* (KACC 12419), *Lactobacillus casei* (KACC 12413) and *Ruminococcus productus* (KCTC 3695).

#### Analysis of microbial community fingerprints and sequencing.

Similarities between the DNA fingerprints were detected using Fingerprinting II software (Bio-Rad). Clustering was performed using the Dice similarity coefficient (*D*_sc_) and UPGMA. The general biodiversity of the Shannon index (*H′*) and the concentration of the dominance or Simpson index (*D* or *S*) were calculated based on the number of bands and their relative intensities among the individual samples. Distinct bands (total of 40) were excised, amplified by PCR with primers 341F (without the GC clamp) and 907R, purified with an *AccuPrep* PCR purification kit (Bioneer) and sequenced (Macrogen). The mean, sd and sem were calculated for each experiment. Student’s *t*-test (unequal variances, two-tailed) was applied for statistical analysis and *P *values <0.05 were considered significant. Multivariate statistics was performed using the supervised principal component analysis (PCA) using simca-p^+^ version 12 (Umetrics).

#### Phylogenetic analysis.

The sequences obtained were checked using ChromasPro (Technelysium) and Sequence scanner version 1.0 (Applied Biosystems) to remove redundant sequences. The partial 16S rRNA gene sequences were compared with those in GenBank (www.ncbi.nlm.nih.gov), the Ribosomal Database Project II (http://rdp.cme.msu.edu) and Eztaxon (www.eztaxon-e.org). Phylogenetic trees were constructed based on the neighbour-joining method by performing 1000 bootstrap analyses using mega4.

#### Quantitative real-time PCR (qPCR).

The qPCR mixture (25 µl) consisted of 1× SYBR *Premix Ex Taq* II (Takara), ROX reference dye II, 0.18 µM each group-specific primer, 2 mM MgCl_2_ and 10 ng DNA template. The amplification programme comprised an initial denaturation step at 95 °C for 30 s, followed by 35 cycles of denaturation at 95 °C for 15 s, primer annealing at 55–68 °C (Table 2) for 20 s and extension at 72 °C for 45 s. The fluorescence data were collected at the end of each PCR cycle and experiments were carried out in triplicate using a 7500 Fast Real-time PCR system (Applied Biosystems). Melting-curve analysis was carried out to determine the specificity of the PCR products. The melting curves were obtained by slowly heating the sample from 60 to 95 °C at a rate of 0.1 °C s^−1^, with continuous fluorescence collection. The fluorescence was compared with the external standard, and the efficiency of amplification of each primer was tested with the appropriate positive and negative controls. The type strains were used to construct the standard curve and the results were converted into the log value of the target bacterial 16S rRNA gene copy numbers (g faecal sample)^−1^.

**Table 2.** List of primers and PCR conditions used for quantification of faecal bacteria using qPCR

Target group*	Sequence (5′→3′)	Size (bp)	Annealing temp (°C)	Mg^2+^ (millimoles l^−1^)	Reference
Universal bacteria†	F: ACTCCTACGGGAGGCAG	468	60	2	Zwielehner *et al.* (2009)
	R: GACTACCAGGGTATCTAATCC				
*Bacteroides–Prevotella–Porphyromonas*‡	See Table 1	140	68	2	Rinttilä *et al.* (2004)
Bifidobacteria§	See Table 1	125	60	2	Zwielehner *et al.* (2009)
*Clostridium coccoides*||	F: CGGTACCTGACTAAGAAGC	429	55	2	Rinttilä *et al.* (2004)
	R: AGTTTYATTCTTGCGAACG				
Lactobacilli¶	F: AGCAGTAGGGAATCTTCCA	341	58	2	Walter *et al.* (2001)
	R: CACCGCTACACATGGAG				

F, Forward; R, reverse.

* The following type strains were used as positive controls: †, *Enterococcus faecalis* (KACC 13807); ‡, *Bacteroides vulgatus* (KCTC 2639); §, *Bifidobacterium longum;* (KACC 20597); ||, *R. productus* (KCTC 3695); ¶, *L. acidophilus* (KACC 12419).

#### Faecal metabolite extraction and derivatization.

Faecal samples (0.1 g) were homogenized with 300 µl methanol (4 °C) for metabolomic analysis. The samples were vortexed for 1 min, ultrasonicated for 30 min and vortexed again for 1 min prior to centrifugation at 16 000 ***g*** for 15 min at 4 °C. Faecal extracts, which were derivatized in two steps to protect carbonyl functions, were dried in a freezing dryer (12 h). First, the dried samples were dissolved in 100 µl of a solution of methoxyamine hydrochloride in pyridine (20 mg ml^−1^; Sigma) and incubated at 75 °C for 30 min. The volatility of polar compounds was increased by exchanging acidic protons against the trimethylsilyl group using 100 µl *N*-methyl-*N*-trimethylsilyltrifluoroacetamide (Sigma) at 70 °C for 30 min.

#### GC-MS-based metabolite profiling.

Each 1 µl of the derivatives were injected in a split mode (1 : 25) into a Varian CP-3800 GC system coupled with a Varian 4000 ion-trap electron ionization/chemical ionization mass spectrometric detector system. A VF-1MS capillary column (30 m×0.25 mm) with 0.25 µm coating and equipped with an integrated 10 m guard column (Varian) was used to separate the derivative metabolites. The initial oven temperature was held at 100 °C for 2 min and then ramped to 300 °C at a rate of 5 °C min^−1^ and held for 7 min. Helium (purity >99.999 %) was used as a carrier gas at a constant flow rate of 1 ml min^−1^. The temperatures of the electron ionization ion source and injector were set to 200 and 250 °C, respectively. Electron impact ionization (70 eV) was utilized, and mass data were collected in a full-scan mode (*m*/*z* 50–1000). The metabolites were identified by comparison with the nist 2005 database version 2.0 (FairCom).

#### Metabolite data analysis.

The raw GC-MS data were converted into CDF (NetCDF) files and processed using the xcms toolbox for automatic peak detection and alignment. For multivariate statistical analysis, the xcms output was further processed using Microsoft Excel. The data were arranged on a three-dimensional matrix consisting of arbitrary peak index (retention time–*m*/*z* pairs), sample names (observations) and peak area (variables).

The resulting three-dimensional data table was entered into the simca-p+ version 12.0 software package for multivariate statistical analysis. The supervised PLS-DA model was used to maximize metabolite variations and identify significantly altered metabolites responsible for such variations (Gao *et al.*, 2009). These values were further validated by Student’s *t*-test using statistica version 7.0 (StatSoft). The metabolites with variable importance projection (VIP) values >1.0 and *P *values <0.05 (threshold) were selected as metabolites that could discriminate between IBS and nIBS patients.

## Results

### Faecal microbial community

PCR amplification and the DGGE profile of total bacteria of the representative faecal samples had a band similarity coefficient of >95 %. Thus, these samples were determined to be basically identical, and the individual sample aliquots were pooled together and used. Significant differences were observed in the banding pattern between IBS (predominant number of bands: 16.9±3.8) and nIBS (9.9±3.5) subjects (*P*<0.001) as well as between the faecal samples. The DGGE pattern showed that diarrhoea-predominant and constipation-predominant faecal samples were mixed in the IBS samples. The intensity of the bands was approximately 42 % higher in the IBS subjects than in the nIBS subjects.

#### Diversity and dominant bacteria.

**Fig. 1.**
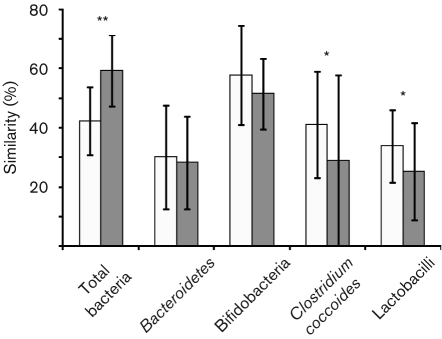
Dice coefficient pairwise comparisons of total bacteria (using universal bacterial primers) and *Bacteroidetes*, *Bifidobacterium*, *Clostridium coccoides* and *Lactobacillus* groups in the IBS versus nIBS faecal community. *, *P*<0.05; **, *P*<0.001. White bars, nIBS; grey bars, IBS.

**Fig. 2.**
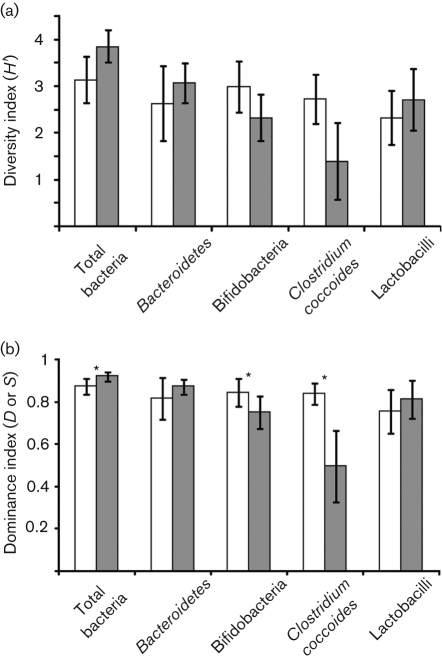
Diversity indices derived from DGGE fingerprinting of 16S rRNA gene-coding regions for total bacteria and the *Bacteroidetes*, *Bifidobacterium*, *Clostridium coccoides* and *Lactobacillus* groups. (a) Shannon index of general diversity (*H′*). (b) Simpson index of dominance (*D* or *S*). *, *P*<0.05; **, *P*<0.01. White bars, nIBS; grey bars, IBS.

**Fig. 3.**
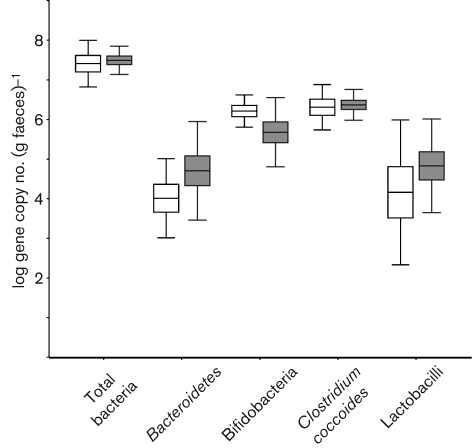
Box and whisker plot showing the quantitative estimation of universal bacteria and group-specific bacteria determined by qPCR. IBS samples were compared with nIBS samples and the values were expressed as the mean log 16S rRNA gene copy number (g faeces)^−1^. The plot displays the following: mean (line inside the box; its position away from the centre represents the degree of skewness in the data), sem (±1 times large box) and sd (‘whiskers’ extending from the upper and lower edges). White bars, nIBS; grey bars, IBS.

**Fig. 4.**
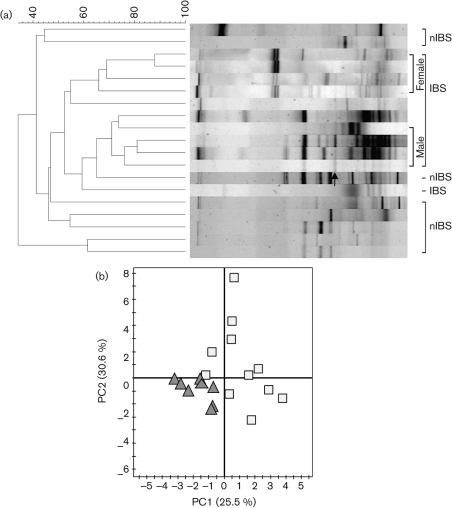
(a) Dendrogram showing the DGGE profiles of the IBS and nIBS faecal bacterial communities. Community DNA was detected by PCR amplification with universal bacterial 16S rRNA gene (V3–V5 regions) primers followed by DGGE. The dendrogram was constructed using *D*_sc_ and the UPGMA algorithm. The arrow indicates the eluted band of *Eubacterium biforme*. (b) PCA of DGGE fingerprints of the 16S rRNA gene of dominant bacteria in IBS (□) and nIBS (▴) samples.

DGGE profiles were used to calculate similarities between the samples and with the selected groups. *D*_sc_ was calculated and clustered, and each sample was compared with the other samples present in the same subject. The mean similarity indices of total bacteria revealed that greater biological variability of predominant bacteria was higher in the IBS samples (59.3±12 %) than in the nIBS samples (42.2±11.4 %) (Fig. 1). The statistical analysis also indicated a significant difference (*P*<0.001) between the IBS and nIBS subjects. The highest microbial diversity (3.36<*H*′<4.37, *P* = 0.004; Fig. 2a) and dominance (0.88<*D*<0.947, *P*<0.05; Fig. 2b) were observed in the IBS sample. In the qPCR analysis, no significant difference (*P*>0.05) in the total quantity was found (Fig. 3). Three main clusters were observed in the DGGE profile, where 90 % of the IBS samples were clustered together. Among the IBS samples, most of the males and females were clustered separately (Fig. 4a). The diverse dataset was grouped based on the variance, and nIBS samples were separated from IBS samples in the first two principal components (PC1 and PC2), which were approximately 26 and 31 %, respectively (Fig. 4b).

The majority of the dominant band sequences (52.5 %) displayed >97 % similarity with known sequences in the database. The most abundant bacterial groups detected were members of the *Firmicutes*, *Proteobacteria*, *Actinobacteria* and *Bacteroidetes* (see Supplementary Fig. S1 with the online journal). Among the dominant bands, 86 % (12/14) had >98 % similarity with the known sequences in the database and were found in both of the study groups. Fingerprinting analysis showed that *Eubacterium biforme* was present exclusively in the IBS subjects (Fig. 4a). The band intensity of *Eubacterium biforme* varied among the samples (2.5–25 %) and its relative quantity varied from 1.3 to 9.5 % among the individual faecal samples.

#### The predominant *Bacteroidetes* (*Bacteroides–Prevotella–Porphyromonas*) group.

The overall gene diversity was 15 % higher in the IBS samples than in the nIBS samples (Fig. 2a). Based on qPCR analysis, the content of *Bacteroidetes* was 15 % higher in the IBS samples than in the nIBS samples. *Bacteroidetes*-specific DGGE profiles were not consistently observed among the subjects and they varied dramatically between samples as well as between the two groups. As the diversity was sample specific, 64 % of the IBS and 50 % of the nIBS subjects were grouped into mixed clusters. In the PCA analysis, nIBS was grouped into PC1 (51 %). Different species of *Prevotella* were identified with >96 % identity using the database. *Bacteroides xylanisolvens* was identified in clustered IBS samples (64 %), with a relative concentration ranging from 7.5 to 20 % in the individual samples. *Bacteroides fragilis* was mostly associated with the male IBS samples (4–20 %).

#### *Lactobacillus* group.

The high *D*_sc_ values suggested that all the nIBS subjects shared a major portion of the band set, whilst the IBS samples had unique bands, as indicated by the low *D*_sc_ values (Fig. 1). The *Lactobacillus* diversity was higher in the IBS samples than in the nIBS samples (Fig. 2). Similarly, the total quantity was about 15 % higher in the IBS subjects than the nIBS subjects. The variances of the IBS subjects were grouped by PC1 (30 %). *Lactobacillus ruminis* was commonly identified in more than half of the IBS samples and had a relative abundance of 5–8 % in the individual IBS samples and a 14–31 % mean band intensity. *Weissella confusa* was widely distributed in all the faecal samples, but a higher band density was observed in the nIBS (57 %) than the IBS (43 %) samples.

The PCR-DGGE fingerprint showed that the mean number of bifidobacteria was higher in the nIBS samples (75 %) than the IBS samples. A similar trend was observed for its diversity (22 %; *P*<0.05) but not in its dominance (11 %; *P*<0.05) for the nIBS samples (Fig. 2a, b). *Bifidobacterium* *pseudocatenulatum* was commonly observed in the faecal samples and had a relative abundance of 6–31 % in the individual samples. The variation in mean band intensity was 60 and 40 % in the IBS and nIBS samples, respectively. *Bifidobacterium* *breve*, *Bifidobacterium* *longum* subsp. *infantis* and *Bifidobacterium pseudolongum* subsp. *pseudolongum* were also observed in the faecal samples.

#### *Clostridium coccoides* group.

*Clostridium coccoides* and related species were detected in all the faecal community DNA. In the analysis of *D*_sc_, a mean of 29 and 40.9 % was determined for the IBS and nIBS samples, respectively, where the latter was 29 % higher, which was numerically significant (*P*<0.05) (Fig. 1). The Shannon index reflected the complete microbial community, and a mean value of 2.73 was observed in the nIBS samples, which was twice as large as the value for the IBS samples (*P* = 0.007; Fig. 2a). The Simpson index of dominance was also 41 % higher in the nIBS sample (*P*<0.05; Fig. 2b). However, there was no difference between the subjects in terms of the total quantity of bacteria. *Blautia wexlerae* (about 94 % similarity) was commonly found in most of the samples (IBS, 73 %; nIBS, 78 %) and constituted up to 48 % among the individual samples.

### Non-targeted metabolite analysis of the faecal extracts

**Fig. 5.**
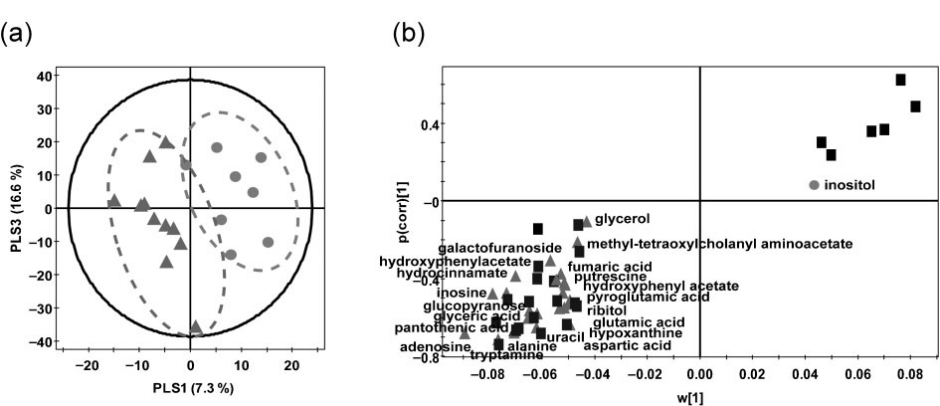
PLS-DA score plots (a) and PLS-DA loading S-plots (b) of significantly different metabolites derived from GC-MS analysis datasets of faecal extracts. (a) •, nIBS; ▴, IBS. (b) •, nIBS-related biomarkers; ▴, IBS-related biomarkers; ▪, unidentified biomarkers.

A total of 1313 peaks were observed when the metabolite profile of the faecal extracts was analysed by xcms from the GC-MS ion chromatograms. The significant differences between IBS and nIBS samples were identified using the mass peak intensities of all the detected metabolites, which were expressed in a score plot for PLS-DA (Fig. 5a) and loading S-plots (Fig. 5b). Both upregulation and downregulation of metabolites were observed in IBS versus nIBS samples. The results are summarized in Supplementary Table S2 (available with the online journal).

### Metabolic signature of IBS and gut microbiota

**Fig. 6.**
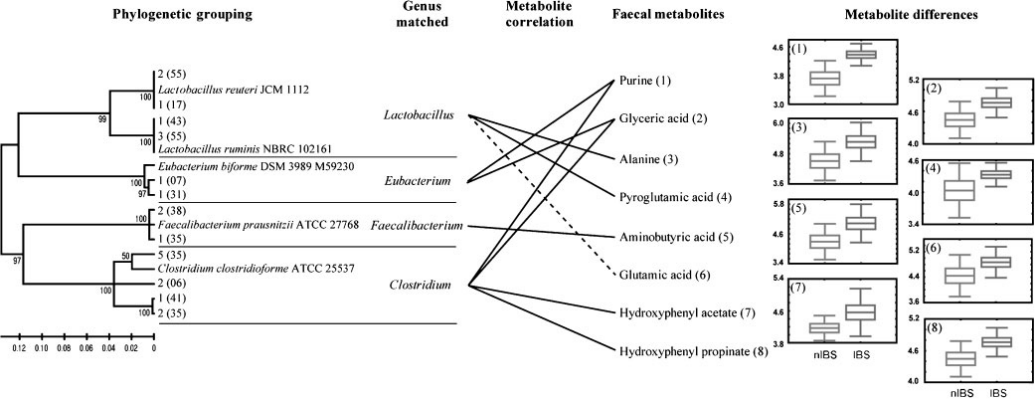
Hypothetical diagram showed a comparison of the faecal metabolites found and the major gut microbiota. The relative changes in IBS and nIBS faecal metabolites were derived from the GC-MS peak area and expressed as box and whisker plots. Positive and negative correlations are indicated as solid and dashed lines, respectively.

The statistical characterization of selected metabolites was related to the identified gut microbiota. Increased levels of amino acids, phenolic compounds, sugar alcohols and purines were observed in IBS faecal samples when compared with nIBS controls. The IBS faecal samples clearly showed higher levels of alanine, pyroglutamic acid, aminobutyric acid, glutamic acid, hydroxyphenyl acetate, hydroxyphenyl propionate, glyceric acid and purines than those in nIBS (Fig. 6). In addition, significantly different metabolites (*P*<0.05) were observed in IBS, including glucopyranose and hydrocinnamic acid; however, these metabolites were not comparable to the currently available gut microbiota due to insufficient literature on gut bacteria and its metabolites. These differences in metabolites between IBS and nIBS faeces were highly correlated with changes in the gut microbiota and provide powerful evidence of the microbial diversity in IBS patients.

## Discussion

The microbiota play a crucial role in the maintenance and regular function of the GI ecosystem. GI microbial communities are stable over a period of time in healthy people with a normal diet; moreover, the population of bacteria depends on individual subjects. Recent studies have shown that there is a high prevalence of IBS in Asians, which is comparable with the levels found in Western populations.

Analysis of the faecal bacterial diversity suggested that the species in the IBS samples were evenly distributed. The mean similarity between the subjects, as well as in the nIBS cases, was low (<50 %), in agreement with published reports (Codling *et al.*, 2010). Three variable regions (V3–V5) of the 16S rRNA gene produced higher variability in the DGGE profile, which implied a low similarity. In contrast, V3-specific amplicons showed the greatest mean similarity (59.2 %) in the faecal bacterial community in Korean samples (Nam *et al.*, 2008). The Dice similarity and UPGMA analyses of the total bacterial DGGE profiles indicated that the nIBS samples were clustered into two groups.

*Firmicutes* and *Bacteroidetes* are the most dominant beneficial microbiota present in the normal human GI tract; in addition, the latter has been shown to be higher in IBS samples (Krogius-Kurikka *et al.*, 2009). The total *Bacteroides–Prevotella–Porphyromonas* quantity determined in this study was in agreement with previous studies, which have reported a relatively increased abundance of these bacteria in elderly people and in those with infectious colitis (Zwielehner *et al.*, 2009). Faecal carbohydrates and insufficient available nitrogen are the limiting factors for GI bacterial metabolism. *Prevotella* produces toxic materials under limiting nitrogen conditions, which is challenging to the survival of other bacteria. Transcriptional activation studies have emphasized the prevalence of *Bacteroides* sp. in other intestinal disorders such as inflammatory bowel disease (Rehman *et al.*, 2010). *Faecalibacterium prausnitzii* produces aminobutyric acid, which is a crucial energy source for colonic epithelium and may enhance the integrity of the epithelial barrier and modulate the GI immune system (Lara-Villoslada *et al.*, 2006). Butyrate has also been reported to modulate inflammation in inflammatory bowel disease patients, possibly by downregulating the production of pro-inflammatory cytokines. *F. prausnitzii* might be important not only for its provision of butyrate to the host but also due to the release of high interleukin (IL-10/IL-12) cytokine levels (Schwiertz *et al.*, 2010).

In the present study, *Eubacterium biforme* (belonging to *Clostridium* cluster XVI) was identified only in IBS subjects. It has been observed in the mucus of patients with Crohn’s disease and in lymphonodular hyperplasia of children (8.33 %) (Harrington *et al.*, 2008). The high level of the sugar alcohol glyceric acid could have been released from triacylglycerols associated with the colon wall; in addition, the increased level of *Eubacterium biforme* in IBS may be responsible for the release of glyceric acids in the faeces, which indicates a shift in energy metabolism.

The genus *Clostridium* consists of a heterogeneous group of micro-organisms that can adapt to diverse habitats (Woodmansey *et al.*, 2004). The Simpson index of dominance was higher in nIBS samples, which is in contrast to other intestinal diseases, and it was not found to be significantly different between Crohn’s disease and control groups (Scanlan *et al.*, 2006). A similar instability of the *Clostridium* group has been observed, and no inter-individual differences between IBS samples were noted (Codling *et al.*, 2010). Direct sequencing has demonstrated that the loss of normal anaerobic bacteria is responsible for the reduction in microbial diversity in IBS (Ott *et al.*, 2004).

Bifidobacterial diversity experiments have shown that IBS samples are clustered separately from the higher diversity of nIBS subjects. The normal dietary fibre-consuming group contained more bifidobacteria than the group consuming less fibre (Benno *et al.*, 1989). A decrease in the amount of dietary fibres consumed led to a reduction in bifidobacteria, which caused constipation in patients with IBS. *Bifidobacterium* is a well-documented taxon with regard to its phylogenetic and phenotypic characteristics (Schwiertz *et al.*, 2010). Although the diversity was significantly higher in nIBS, no major difference was observed in terms of total number of bifidobacteria. *Bifidobacterium* *breve* and *Lactobacillus plantarum* probiotic combinations have been reported to reduce the severity of IBS symptoms, and the prebiotics oligofructose and inulin selectively stimulate bifidobacterial growth and reduce constipation, predominant in IBS patients (Saggioro, 2004).

With regard to the *Lactobacillus* group, neither the IBS nor nIBS subjects clustered separately, as the diversity and abundance were higher in the IBS group but lower for the Dice similarity index. Among the selected 20 phylotypes, 3 different genera and 12 species were matched (>97 %) with either the type strains or isolates from fermented foods. Qin *et al.* (2010) reported that the abundance of many bacteria varies greatly within individuals over a period of time and even within the phylum level. It has been recognized that probiotic bacteria help human digestive health. However, modern and advanced tools have improved our understanding of bacterial niches and the influence of metabolites. It is understood that the colonic environment has different nutritional patterns and microbial ecosystems, which has a relationship with metabolic niches. The increased level of faecal d-alanine produced by lactic acid bacteria modulates the immune system in a colitis animal model (De Vos, 2005). The elevated levels of purine in faecal metabolites suggested extensive glycogenolysis and glycolysis; it also indicated increased microbial activity in either the proliferation or degradation of bacterial cells. In contrast, qPCR revealed that the quantity of lactobacilli was lower in nIBS patients than in the normal gut, but it was higher in IBS patients, and it has been suggested that lactobacilli and the secreted organic acids may cause IBS symptoms (Tana *et al.*, 2010). The low level of alanine and pyroglutamic acid in nIBS indicates lowered bacterial protease and peptide catabolism, whereas *Lactobacillus* activity was increased in IBS, as evidenced by the elevated levels of faecal alanine released by protein catabolism (Romick-Rosendale *et al.*, 2009). GI tract proteins are partially hydrolysed as a result of the acidic conditions of the GI tract and produce peptides, which are then broken down by gut microbial proteases to release amino acids (Romick-Rosendale *et al.*, 2009). There are two possible reasons for the elevated levels of faecal amino acids: one is catabolism of peptides by the higher bacterial load; the second is malabsorption of amino acids in IBS patients. The elevated level of gut bacteria is also reflected in energy metabolism changes, thus stimulating gut carbohydrate metabolism. When the carbohydrates and free amino acids are replaced by protein/peptides, the fermented phenolic compounds were increased in the faeces. Lactobacilli produce pyroglutamic acid, bacteriocin, lactic acid and acetic acid, which act as antimicrobials and also lower the pH levels, thus preventing the colonization by other bacterial communities (Engberg *et al.*, 2000; Salonen *et al.*, 2010). Fermentation of tyrosine by proteolytic bacteria ends with phenol products. This metabolic signature is similar to that observed in conditions of stress and trauma under the influence of human physiological changes.

The low level of alanine and pyroglutamic acid in nIBS indicates a lowered level of bacterial protease and peptide catabolism, whereas *Lactobacillus* activity is increased in IBS, indicated by the elevated levels of faecal alanine released by protein catabolism (Romick-Rosendale *et al.*, 2009). The IBS samples displayed higher levels of hydroxyphenyl acetate and hydroxyphenyl propionate, which are converted from phenylalanine and plant phenolic compounds by *Clostridium* spp. as intermediates (Winter *et al.*, 1991). The increased level of aminobutyric acid in IBS was strongly correlated with the presence of *F. prausnitzii*, which was the most significant *n*-butyrate-producing gut bacterium, with well-known effects on host energy metabolism and mucosal integrity (Li *et al.*, 2008; Pryde *et al.*, 2002).

In conclusion, we systematically examined the microbial community and metabolites in faecal samples of IBS and nIBS Korean subjects. Our results demonstrated a clear discrepancy between the faecal microbial diversity and richness measured as the presence of 16S rRNA gene signatures of bacteria and their metabolic activities. Key bacterial communities were shown to be significantly different in diversity and dominance between IBS and nIBS samples. The diversity and quantity were inter-linked only in the *Bacteroidetes*, *Bifidobacteria* and *Lactobacillus* groups. The higher levels of amino acids and phenolic compounds indicated increased bacterial metabolism and also altered energy by the predominant gut microbiota.

## Supplementary Material

Supplementary Data
