# Supplementary material for: Microbial community and metabolomic comparison of irritable bowel syndrome faeces
Source: J Med Microbiol. 2011 Jun;60(Pt 6):817–27. doi: 10.1099/jmm.0.028126-0 (PMC3167923; doi:10.1099/jmm.0.028126-0)
Supplement: Supplementary Data [file supp_60_6_817__index.html]

Supplementary Data 

# Microbial community and metabolomic comparison of irritable bowel syndrome faeces

### Microbial community and metabolomic comparison of irritable bowel syndrome faeces, by K. Ponnusamy, J. N. Choi, J. Kim, S.-Y. Lee and C. H. Lee

*Journal of Medical Microbiology* vol. **60**, part 6, pp. 817 - 827

**Supplementary Data** [PDF file] (32 KB)

**Supplementary Fig. S1**  
 Neighbour-joining tree showing the phylogenetic relationship of DGGE derived from universal bacterial 16S rRNA gene (primers 341F/907R) sequences. Isolates that showed 99 % identity were grouped on the same line. Sequences obtained in the present study are represented with the DGGE band number; the sample ID is provided in parentheses and the eluted *Eubacterium biforme* bands are indicated in bold. Phylum names given on the right are based on the taxonomic hierarchy used in the second edition of *Bergey�s Manual of Systematic Bacteriology*. Accession numbers of the type strain sequences retrieved from GenBank are given together with their names. The bootstrap values on the nodes are percentage confidence levels of 1000 replications.

**Supplementary Table S1**  
 Demographic and clinical characteristics of IBS and nIBS samples.

**Supplementary Table S2**  
 Significantly different metabolites between IBS patients and nIBS controls identified by GC-MS.
