# Supplementary material for: Microbial community and metabolomic comparison of irritable bowel syndrome faeces
Source: J Med Microbiol. 2011 Jun;60(Pt 6):817–27. doi: 10.1099/jmm.0.028126-0 (PMC3167923; doi:10.1099/jmm.0.028126-0)
Supplement: Supplementary Data [file supp_60.6.817_JMM_28126_48612_Suppl_data_Fig_S1_Tables_S1-2.pdf]

Supplementary Fig. S1

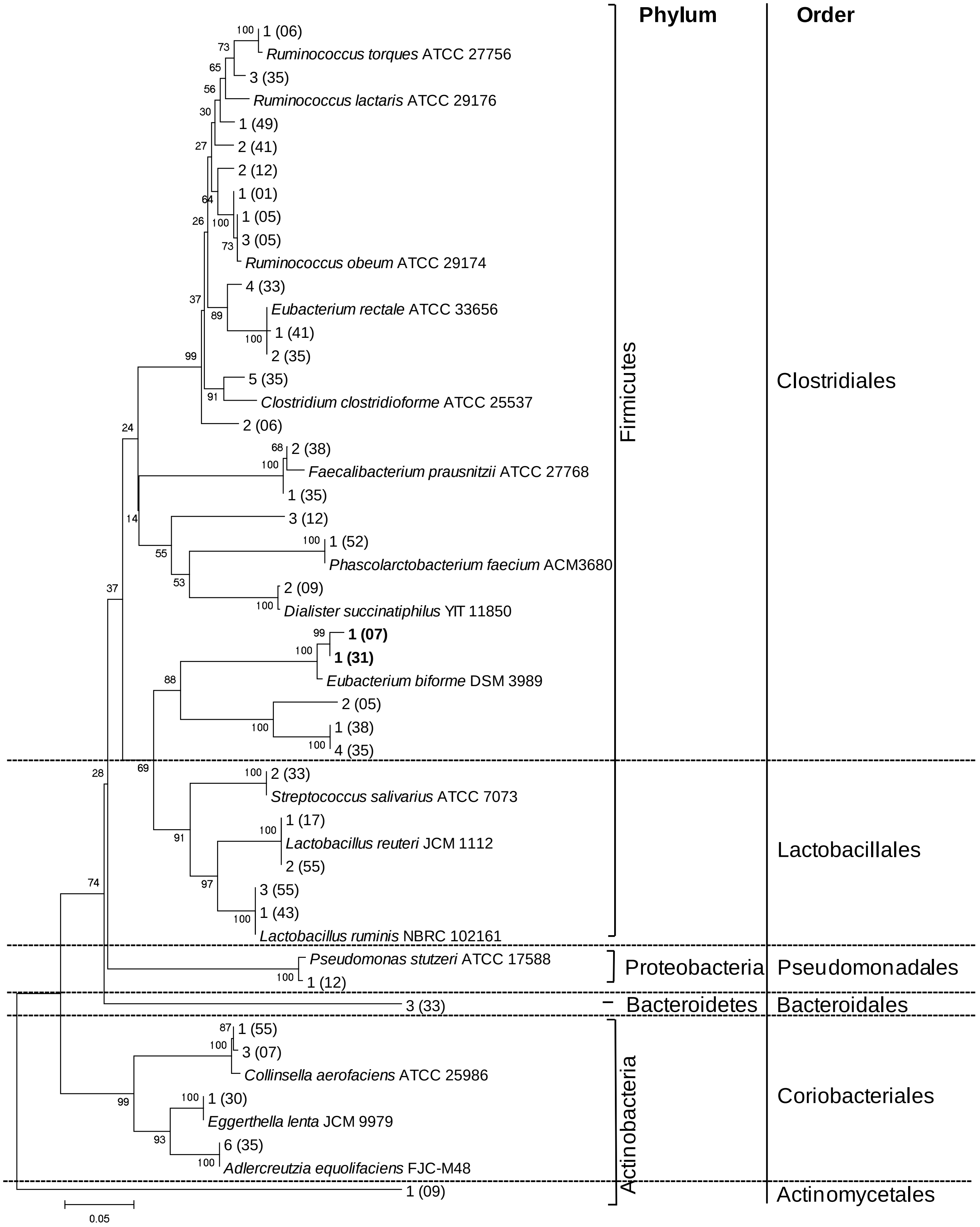

Supplementary Table S1. Demographic and clinical characteristics of IBS and nIBS samples

Sample ID	Sex	Age (years)	Remarks
IBS 01	F	46	DP
nIBS 05	M	68	–
nIBS 06	F	26	–
IBS 07	F	74	CP
IBS 09	F	63	–
IBS 12	M	64	MT
nIBS 15	F	44	–
IBS 17	M	66	DP, rectal cancer
IBS 22	M	62	DP
IBS 30	F	43	CP
IBS 31	M	34	DP
nIBS 33	M	60	–
IBS 35	M	48	MT
nIBS 38	M	25	–
nIBS 41	M	26	–
nIBS 43	M	32	–
nIBS 49	M	53	–
IBS 52	F	18	DP
IBS 55	M	51	DP

CP, Constipation predominant; DP, diarrhoea predominant; F, female; M, male; MT, mixed type.

Supplementary Table S2. Significantly different metabolites between IBS patients and nIBS controls identified by GC-MS

Separation is based on a VIP analysis with a cut-off value of 1.5.

Variable no.	Retention time (min)	Fragmentation	Name	Trimethylsilylation	*P* value*
45	9.25	292, 189, 184, 147, 134, 103, 73	Glyceric acid	×3	0.03
33	9.39	245, 217, 147, 98, 83, 73	Fumaric acid	×2	0.10
87	11.38	290, 248, 174, 147, 133, 100, 86, 73	Alanine	×3	0.04
1155	13.24	258, 230, 156, 147, 73	Pyroglutamic acid	×2	0.10
706	13.45	349, 306, 232, 218, 202, 188, 147, 133, 100, 73	Aspartic acid	×3	0.09
47	13.68	304, 246, 216, 174, 147, 100, 86, 73, 59	Aminobutyric acid	×3	0.05
1239	15.54	348, 246, 230, 147, 128, 100, 84, 73	Glutamic acid	×3	0.10
148	15.62	296, 281, 252, 179, 164, 103, 73	Hydroxyphenyl acetate	×2	0.10
4	17.51	310, 295, 205, 192, 177, 163, 147, 73	Hydroxyphenyl propionate	×2	0.02
14	18.16	310, 295, 192, 179, 163, 73	Hydrocinnamic acid	×2	0.01
27	18.24	319, 217, 204, 47, 103, 73	Galactofuranoside	×4	0.07
505	18.41	361, 214, 200, 174, 73	Putrescine	×4	0.10
173	19.01	280, 265, 238, 206, 147, 73	Hypoxanthine	×2	0.10
159	22.64	421, 331, 319, 305, 217, 205, 157, 147, 129, 73	Dulcitol	×6	0.08
64	23.08	420, 291, 246, 201, 159, 117, 73	Pantothenic acid	×3	0.02
18	26.91	361, 273, 202, 174, 86, 73	Tryptamine	×3	0.01
7	32.46	541, 395, 281, 259, 245, 217, 193, 169, 147, 103, 73	Inosine	×4	0.01
1	33.37	540, 450, 281, 245, 236, 230, 217, 147, 117, 73	Adenosine	×4	0.00
28	34.21	437, 361, 319, 271, 243, 217 193, 169, 147, 103, 73	Glucopyranose	×8	0.03

*Wilcoxon rank test.

Ponnusamy, K., Choi, J. N., Kim, J., Lee, S.-Y. and Lee, C. H. (2011). Microbial community and metabolomic comparison of irritable bowel syndrome faeces. *Journal Med Microbiol* **60**, 817–827.
